# Design of a tailor‐made platform for syngas bioconversion into polyhydroxybutyrate

**DOI:** 10.1111/1751-7915.12847

**Published:** 2017-08-25

**Authors:** Tanja Narancic, Kevin E. O'Connor

**Affiliations:** ^1^ UCD Earth Institute and School of Biomolecular and Biomedical Science University College Dublin Belfield, Dublin 4 Ireland; ^2^ BEACON – Bioeconomy Research Centre University College Dublin Belfield, Dublin 4 Ireland

## Abstract

Biodegradable polymers such as polyhydroxybutyrate (PHB) are part of the emerging portfolio of renewable materials, which are addressing the issue of plastic waste. Syngas, as a cheap, renewable and sustainable resource that can be obtained from biomass or waste, is viewed as an excellent feedstock for different bioprocesses, including syngas to PHB bioconversion. However, due to the hazardous nature of syngas, it is of utmost importance to consider safety aspects of the process. This recently developed tailor‐made platform for safe syngas fermentation and PHB production addresses safety aspects and demonstrates the importance of robust online and in‐line analytical tools allowing for monitoring and controlling of this bioprocess.

## Abstract 2

Biodegradable polymers such as polyhydroxybutyrate (PHB) are part of the emerging portfolio of renewable materials, which are addressing the issue of plastic waste. Syngas, as a cheap, renewable and sustainable resource that can be obtained from biomass or waste, is viewed as an excellent feedstock for different bioprocesses, including syngas to PHB bioconversion. However, due to the hazardous nature of syngas, it is of utmost importance to consider safety aspects of the process. This recently developed tailor‐made platform for safe syngas fermentation and PHB production addresses safety aspects and demonstrates the importance of robust on‐line and in‐line analytical tools allowing for monitoring and controlling of this bioprocess.

Municipal solid waste (MSW) is a complex mixture of a wide range of materials we use and throw away on a daily basis. The level of produced MSW varies from country to country, and it can be as low as 286 kg per capita in Poland to 789 kg per capita in Denmark in 2015 (Eurostat, 2015). MSW generates a lot of attention because of its high complexity and links to consumption patterns (Eurostat, 2015). The challenge we face is to ensure that the economic growth, which provides jobs and general well‐being, leads to a sustainable future. Therefore, the strategy of the EU and the USA is to reduce the proportion of MSW deposited in landfills from currently, 31% and 53%, respectively (EC, 2015; EPA, 2015). The waste management options include recycling and reuse of the waste. Despite the 2.6‐fold increase in recycling between 1995 and 2015, the share of recycled municipal waste is still only 29% (Eurostat, 2015), due to the complexity of the waste, pre‐treatments and transport. However, treating MSW as a resource that could be fed back into economy creates great opportunity to improve the waste management and open up new avenues.

Pyrolysis is one of the means to harvest MSW as a resource. In this process of thermochemical decomposition of carbonaceous material, a synthesis gas or syngas is formed. Syngas is predominantly composed of carbon monoxide, carbon dioxide and hydrogen. The potential of syngas is tremendous, as it can directly be used as a fuel, or can be converted to liquid fuels (Beneroso *et al*., 2017). Furthermore, a technology was demonstrated for the microbial conversion of corn seed‐derived syngas into polyhydroxybutyrate (PHB), a polypropylene‐like biopolyester (Do *et al*., 2007). However, industrial‐scale syngas to PHB conversion has not been reported to date, mainly due to challenges associated with the cultivation and PHB productivity of *Rhodospirillum rubrum*, the microorganism used in the process (Karmann *et al*., 2017).

Karmann and co‐workers recently demonstrated a platform set‐up for safe syngas fermentation and PHB production at larger scale, with specifically integrated process analytical technology tools which allow acquisition of maximum information about the bioprocess (Karmann *et al*., 2017). The safety aspects are the imperative of a good laboratory practice. In syngas fermentations, large amounts of highly flammable hydrogen and extremely toxic carbon monoxide are continuously sparged into a bioreactor. Both gases are colourless and odourless and thus difficult to detect. The syngas fermentation platform designed by Karmann *et al*. (2017) consists of a bioreactor placed in a fume hood, while the gas cylinders are stored outside and the gas is transported through pipes to the bioreactor. Additionally, this tailor‐made platform is equipped with hydrogen and carbon monoxide detectors and alarms.

The success of a fermentation process heavily depends on physiological parameters of the organism. Therefore, integration of quantitative real‐time analytical tools into the fermentation platform allows monitoring and controlling of the process. Carbon monoxide dehydrogenase (CODH), a crucial enzyme for assimilation of carbon monoxide *via* carbon dioxide, is inactive at redox potentials above −300 mV (Heo *et al*., 2001). The addition of an online redox sensor to the syngas fermentation platform therefore provides vital information on the conditions indicating the level of CODH activity. Furthermore, the concentration of dissolved carbon monoxide seems to follow the redox potential trend, and thus information on the real‐time redox potential might indicate a limitation in dissolved carbon monoxide (Karmann *et al*., 2017). Additionally, PHB metabolism is dynamic and affected by environmental conditions (Narancic *et al*., 2016). The syngas fermentation platform designed by Karmann *et al*. (2016, 2017) uses flow cytometry as a robust and fast quantitative tool, which allows adjustment of carbon supply or selection of the appropriate harvesting time in order to maximize PHB production.

Polyhydroxyalkanoates, including PHB, have been in the spotlight for a number of years as materials which could be used to replace conventional plastic materials. This could help reduce or remove the negative impacts of increasing post‐consumer plastic waste as a part of a strategy for environmentally and socially sustainable economy. Furthermore, syngas, which can be obtained from various low‐grade feedstocks (Mahinpey and Gomez, 2016) that are not in competition with the food chain, could be used not only for the production of PHB (Revelles *et al*., 2016; Karmann *et al*., 2017), but for the production of bio‐based chemicals as well (Griffin and Schultz, 2012; de Jong *et al*., 2012). The tailor‐made platform for syngas conversion into PHB could therefore be used to expand the conversion of syngas into other value‐added products *via* microbial fermentation.

## Conflict of Interest

None declared.
